# A New Series of Kinked Liquid Crystals: 2-(6-Alkoxynaphthalen-2-yl)-6-methoxyquinolines

**DOI:** 10.3390/ijms16047520

**Published:** 2015-04-02

**Authors:** Win-Long Chia, Chih-Wei Liu

**Affiliations:** Department of Chemistry, Fu Jen Catholic University, New Taipei City 24205, Taiwan; E-Mail: peter19871221@me.com

**Keywords:** liquid crystals, kinked mesogen, synthesis, thermotropic behavior

## Abstract

A new series of 2-(6-alkoxynaphthalen-2-yl)-6-methoxyquinolines (*n*O-NpQOMe, *n* = 3–8) liquid crystal compounds, a linear molecular structure with two kinks, were synthesized using a short two-step reaction with overall yields between 43% and 58%. Spectral analyses were in accord with the expected structures. Thermotropic behavior of these liquid crystal compounds were investigated using polarized optical microscopy and differential scanning calorimetry. All compounds exhibited purely enantiotropic nematic phase at the medium–high temperature range of 162.4–234.2 °C. However, short ranges of nematic phase, 20.5–16.6 °C at heating and 46.7–37.0 °C at cooling, were observed in these linear liquid-crystalline compounds with two kinks.

## 1. Introduction

The nematic phase is technologically the most crucial phase among many liquid crystalline phases. This phase typically appears directly below the isotropic liquid phase, and a liquid in the nematic phase flows like a simple liquid. Although the nematic phase is usually schematically represented as linear molecules aligned uniaxially with their long axes parallel to each other, the structures of nematogenic molecules are not necessarily completely linear. A short tail, a short core with a conjugative linking group, and a polar end group usually exhibit the nematic phase [1,2]. However, kinked moieties, naphthalene and quinoline, were found to exhibit the nematic phase [3,4,5,6,7]. Therefore, the authors believe that some more subtle structural factors must be clarified for understanding the nematogenity of liquid crystal molecules.

Because synthesizing quinoline-containing liquid crystalline compounds is difficult, detailed comparisons of the trends in their homolog series have rarely been investigated. Quinoline-containing liquid crystalline compounds are typically synthesized through a cyclization reaction, such as the reaction of suitable substituted anilines and benzaldehydes with pyruvic acid, followed by the decarboxylation of the corresponding carboxylic acids [8]. Other quinoline-compounds are synthesized by heating aniline with glycerin, 1,2-glycols, or unsaturated aldehydes by using the Skraup procedure or the Doebner-Von Miller variation [9,10,11,12,13,14]. Few studies have reported the bis-formylation of acetanilides, followed by cyclization with polyphosphoric acid and subsequent conversion to chloroquinoline aldehydes, which are used as intermediates to further synthesize quinoline-containing liquid crystalline compounds [15,16,17]. Although these methods are extremely valuable for constructing crucial quinoline systems, most of them have a limited scope, involve numerous synthesis steps, and produce low yields. However, several recent patents have generated optical-switching elements with a high-speed response by using quinoline-containing liquid crystalline compounds [18,19,20]. Therefore, we decided to develop a new method for synthesizing quinoline-containing liquid crystalline compounds.

We previously reported a novel method for synthesizing pyridine- and quinoline-containing liquid crystalline compounds [21,22,23,24,25,26,27]. These compounds have been attractive synthetic targets because of their chemo- and regioselective reactions, tolerating various functional groups. This paper reports the syntheses and thermotropic behaviors of a new homologous series of 2-(6-alkoxynaphthalen-2-yl)-6-methoxyquinolines (*n*O-PPQOMe), where *n* varies from 3 to 8 (propyl to octyl). The liquid crystal molecules designed in this study contain a linear mesogenic core with two kinks consisting of naphthalene and quinoline moieties, and a polar methoxy end group.

## 2. Results and Discussion

### 2.1. Synthesis

**Scheme 1:**
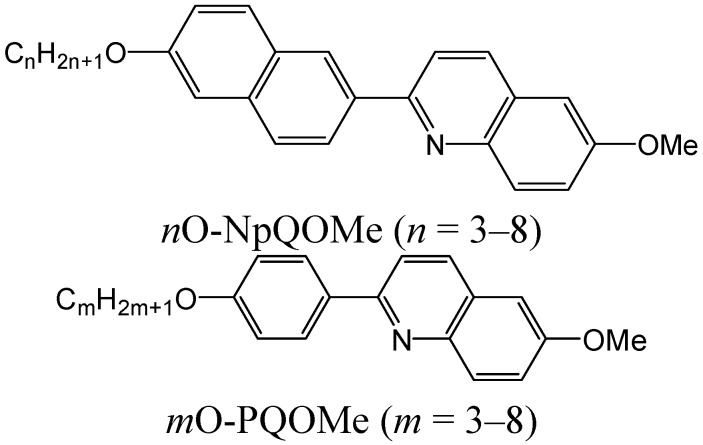

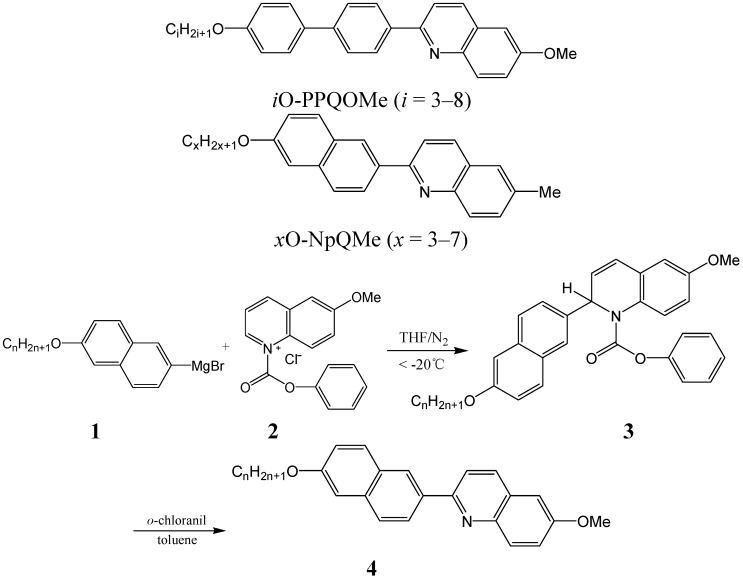
Synthetic route for synthesizing *n*O-NpQOMe.

A new homologous series of *n*O-NpQOMe, *n* = 3–8 was obtained first through the regioselective addition of Grignard reagents to activated 1-acylquinolinium salts to preferentially form 1,2-dihydroquinolines, which were then aromatized through a mild oxidation reaction (Scheme 1).

Grignard reagent **1** was prepared by reacting magnesium with an appropriate 2-bromo-6-alkoxynaphthalene, which was obtained by reacting 2-bromo-6-hydroxynaphthalene with an appropriate bromoalkane. The reaction between 6-methoxyquinoline and phenyl chloroformate produced 6-methoxyquinolinium chloride **2**. The reaction between Grignard reagents **1** and **2** generated the expected 1,2-dihydroquinoline adduct **3**. Adduct **3** was then oxidized using *o*-chloranil to produce the desired *n*O-NpQOMe **4** (*n* = 3–8).

Phenyl chloroformate was used to increase the reactivity of quinoline and facilitate the regioselective nucleophilic attack of the Grignard reagent. This synthetic methodology favored Grignard α-regioselectivity at the quinoline ring [28,29]. Because of the high polarity difference between the major α and minor γ products, trace amounts of γ products were easily separated using liquid chromatography with a 2:1 methylene choloride:hexane eluant system. The yields of this 2-step reaction ranged from 43% to 58% (Table 1). Highly pure products were collected by recrystallizing them several times by using methylene chloride and ethyl acetate.

**Table 1:** Yields of 2-(6-alkoxynaphthalen-2-yl)-6-methoxyquinolines (*n*O-NpQOMe, *n* = 3–8).

Entry (*n*)	Alkyl Group	Yield ^a^ (%)
3	Propyl	58
4	Butyl	43
5	Pentyl	46
6	Hexyl	52
7	Heptyl	52
8	Octyl	48

^a^ Isolated yields are quoted after recrystallization.

### 2.2. Thermotropic Studies

Phase transition temperatures and the associated enthalpy changes of the series of *n*O-NpQOMe compounds were determined using differential scanning calorimetry (DSC). The heating and cooling rates were set to 5 °C·min^−1^. The corresponding mesophases of the *n*O-NpQOMe compounds were identified by observing their textures by using polarized optical microscopy (POM) as shown in Table 2 [30]. Because two consecutive kinked structural moieties (naphthalene, quinoline) existed along the molecular long axis, an enantiotropic nematic phase was the sole mesophase observed in all *n*O-NpQOMe (*n* = 3–8) compounds. The nematic phase was observed at the medium–high temperature range of 162.4–234.2 °C. As for the thermal stability of the *n*O-NpQOMe compounds, the temperature at which five percent of the initial mass was lost was in the range of 278–323 °C for *n* = 3–8.

**Table 2:** Phase transition temperatures (°C) and the corresponding transition enthalpies (kJ·mol^−1^), in parentheses, for the homologous series of *n*O-NpQOMe, *n* = 3–8, compounds.

Compound *n*O-NpQOMe (*n*)	Phase Transition Temperatures (°C) and Corresponding Transition Enthalpies (kJ·mol^−1^)	
	Heating	Cooling
3	Cr 213.7(31.81) N 234.2(0.27) I	I 232.6(0.37) N 185.9(27.13) Cr
4	Cr 205.3(29.51) N 231.6(0.40) I	I 230.1(0.45) N 175.1(25.05) Cr
5	Cr 196.1(25.71) N 217.7(0.25) I	I 216.3(0.28) N 169.3(23.05) Cr
6	Cr 192.1(29.48) N 213.9(0.24) I	I 212.2(0.26) N 167.7(27.23) Cr
7	Cr 187.0(24.95) N 204.4(0.21) I	I 203.0(0.23) N 163.2(21.84) Cr
8	Cr 184.4(24.94) N 201.0(0.19) I	I 199.4(0.27) N 162.4(23.28) Cr

Scan rate: 5 °C·min^−1^; Cr = crystal phase, N = nematic phase, I = isotropic phase.

**Figure 1:**
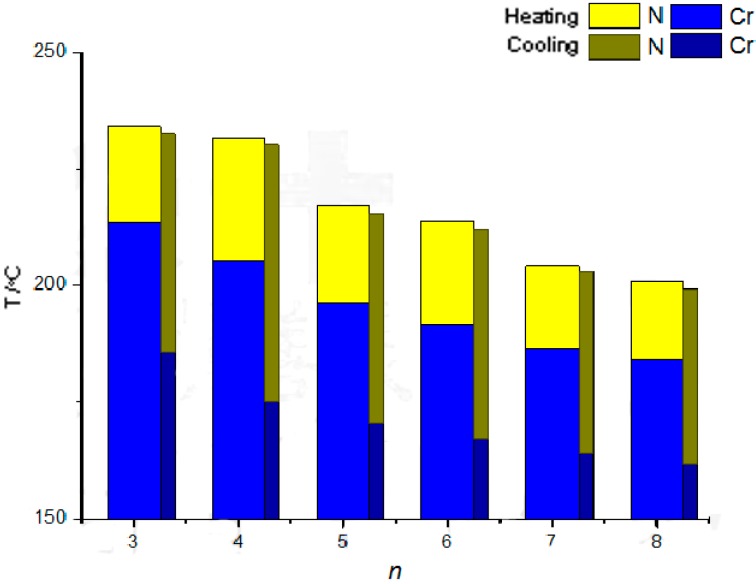
Plot of transition temperatures of the heating and cooling cycle as a function of the terminal alkoxy chain length *n*O-NpQOMe, *n* = 3–8, compounds.

Figure 1 depicts a plot of the transition temperatures of the heating and cooling cycles *vs.* the number of carbons in the terminal alkoxy chain of the *n*O-NpQOMe (*n* = 3–8) compounds. The melting point decreased steadily from 213.7 to 184.4 °C (difference: 29.3 °C) with an increase in the length of the alkoxy chain, whereas the freezing point decreased as a tail in an exponential decay curve from 185.9 to 162.4 °C (difference: 23.5 °C) with an increase in the length of the alkoxy chain. Severe hysteresis were observed in all *n*O-NpQOMe, *n* = 3–8, compounds. Therefore, the nematic phase lengths at cooling (37.0–55.1 °C) were approximately twice those at heating (16.6–26.4 °C). It is known that the melting (freezing) point of an organic compound is dependent upon its molecular weight, molecular symmetry, and thus the compactness of molecules in a three-dimensional lattice structure. Thus, the doubly kinked, asymmetrical mesogenic cores (naphthalene and quinoline) in *n*O-NpQOMe appear to prevent the ease of nucleation during crystallization from mesophase and delay the freezing process.

Similar to the decreasing trend observed for melting points, the nematic-to-isotropic transition temperature, *T_NI_*, decreased steadily from 234.2 to 201.0 °C (difference: 33.2 °C) with an increase in the length of the alkoxy chain. Likewise, the doubly kinked, asymmetrical mesogenic cores are responsible for the collapsing process of the crystalline-to-nematic transition and nematic-to-isotropic transition. The soft alkoxy chain that suppresses the crystallization seems to function as an “impurity” in the crystalline/liquid-crystalline three dimensional lattice structure consisting of anisotropic hard cores.

If the *T_NI_* points are grouped into three pairs (*n* = 3, 4; 5, 6; and 7, 8), then the points in each pair are only slightly different from each other (234.2 and 231.8 °C; 217.7 and 213.9 °C; and 204.4 and 201.0 °C for *n* = 3, 4; 5, 6; and 7, 8, respectively, for heating; similar behavior was observed for cooling). Conventionally, the anisotropy of molecular polarizability is greater for alkoxy chains with an even number of carbons, and their *T_NI_* values are higher than those for alkoxy chains with an odd number of carbons. Therefore, the *T_NI_* points vary in a zigzag manner as *n* increases [31]. However, the *T_NI_* points for alkoxy chains with a lower and odd number of carbons in the series of *n*O-NpQOMe, *n* = 3–8, compounds were slightly higher than those of their paired abutting even members. A possible explanation can be ascribed to the possible dimer formation in the nematic phase and that the proportion ratio of hard core/soft alkoxy chain is higher for those alkoxy chains with a lower and odd number of carbons.

A progressive decrease in the anisotropy of the molecular polarizability of the longer alkoxy homologs was observed in the nematic phase. Damping of the stepwise decrease in *T_NI_* can be explained by a statistical increase in the number of non-extended conformations [31].

The nematic phase lengths are relatively independent of the odd–even effect, and these lengths during the heating and cooling of *n*O-NpQOMe, *n* = 3–8, compounds was maintained in the range of 16.6–26.4 and 37.0–55.1 °C, respectively. The authors believe that the trans-conformation of the two consecutive kinked mesogenic cores dominates and maintains the appearance of the nematic mesophase. Therefore, the nematic phase lengths are relatively independent of the influence of the alkoxy chain lengths.

The enthalpies of nematic-to-isotropic transitions in the series of *n*O-NpQOMe compounds, ΔH_NI_ (or ΔH_IN_), were in the order of 0.19–0.45 kJ·mol^−1^. Although the molecular weights of these *n*O-NpQOMe (*n* = 3–8) compounds are high (343–413 g/mol), low mesomorphic transition enthalpies indicate a loose intermolecular packing in their corresponding nematic phases. If these enthalpies are calculated and expressed in terms of entropy changes, and are scaled by the gas constant, then ΔS_NI_/R is 0.53, 0.79, 0.51, 0.49, 0.44, and 0.40 for *n* = 3–8, respectively [32,33,34,35]. Both the enthalpy and entropy changes associated with the nematic-to-isotropic transition decreased gradually as *n* increased from 4 to 8. In the nematic phase, the shorter the alkoxy chain, the higher its mobility (except for *n* = 3, which is too short to show a high entropy difference in its nematic-to-isotropic transition). Although the flexible tail is responsible for suppressing the crystallization, the attractive forces from the alkoxy chains increase as the alkoxy chain length increases (from *n* = 4 to 8). A smetic phase (another degree of order) usually results in many other series of liquid crystals when *n* ≥ 8. However, the doubly kinked hard core in *n*O-NpQOMe is designed and prepared to suppress further orderliness of molecular spatial arrangement, thus only the nematic phase is observed.

**Figure 2:**
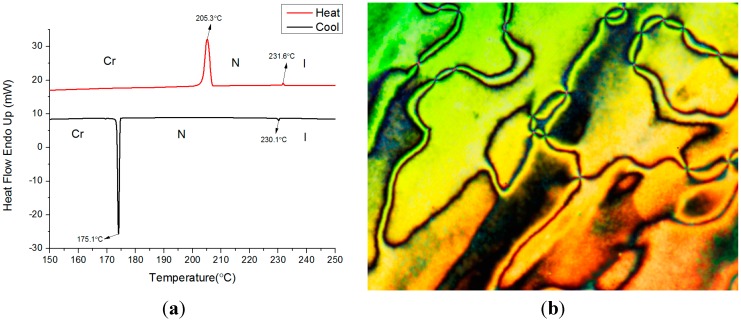
*4*O-NpQOMe (**a**) Thermograms of a second DSC scan at a heating (red) and cooling (black) rate of 5 °C·min^−1^; (**b**) Polarized optical micrograph originating from the isotropic phase on cooling to 240 °C, nematic schlieren texture, ×200.

Figure 2a,b depicts DSC thermograms of *4*O-NpQOMe and its nematic schlieren texture, respectively. During the second scan of the heating process, two endothermic peaks were observed at 205.3 and 231.6 °C, and two exothermic peaks were observed at 230.1 and 175.1 °C during the cooling process. The enantiotropic nematic phase, supercooling phenomenon, and small cusps of the nematic-to-isotropic transition, *T_NI_* (or *T_IN_*), of *4*O-NpQOMe were readily observed in this thermogram. The typical nematic schlieren texture can be identified by the characteristic brushes together with a dark area from the homeotropic alignment of liquid crystal molecules.

A previous study on a series of *m*O-PQOMe, *m* = 3–8, compounds [23] showed that all of these compounds exhibited a monotropic nematic phase in the range of 116.1–148.1 °C, with short phase lengths in the range of 13–24 °C. Compared with *n*O-NpQOMe compounds, the mesogenic core in *m*O-PQOMe, containing phenyl and quinoline moieties, is too short to exhibit a stable enantiotropic nematic phase.

**Figure 3:**
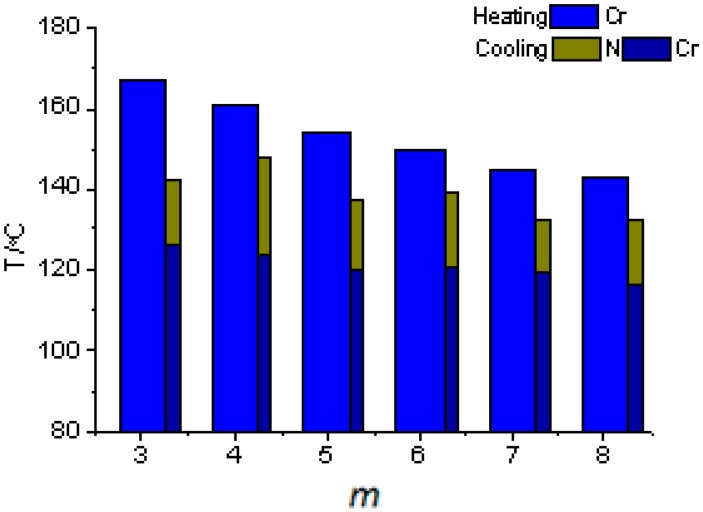
Plot of transition temperatures of heating and cooling cycle as a function of the terminal alkoxy chain length of *m*O-PQOMe, *m* = 3–8, compounds.

In the series of *m*O-PQOMe compounds, *T*_IN_ varied in a zigzag manner in the range 132.6–148.1 °C, and appeared to slightly decay as the alkoxy chain length increased, as shown in Figure 3. The upper points represent an even number of carbons, 148.1, 139.2, and 132.7 °C for *m* = 4, 6, and 8, respectively, and the lower points represent odd number of carbons, 142.6, 137.7, and 132.6 °C for *m* = 3, 5, and 7, respectively [31]. The variation in *T_IN_* exhibits its characteristic feature of odd-even effect regardless of the behavior of the melting and freezing points. Furthermore, the experimental results showed that the alkoxy chains with an even number of carbons have wider nematic lengths than those with an odd number of carbons do. This implies that in the series of *m*O-PQOMe compounds the soft alkoxy chain participates in not only the generation but also the extension of the monotropic nematic phase.

The ΔS_IN_/R values for the series of the *m*O-PQOMe compounds were 0.20, 0.17, 0.12, 0.12, 0.09, and 0.14 for *m* = 3–8, respectively. Again, these low values for the isotropic-to-nematic transitional entropy indicate the closeness of randomness of the *m*O-PQOMe compound between its mesophase and isotropic phase. That is, these low values for the isotropic-to-nematic transitional entropy can be attributed to the increased molecular biaxiality (kinked structure) that originated from the short mesogenic core with phenyl and quinoline moieties.

**Figure 4:**
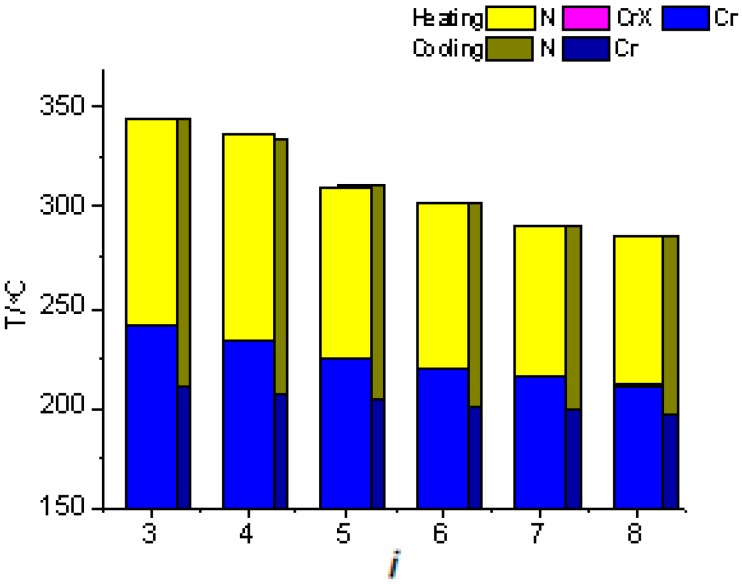
Plot of transition temperatures of heating and cooling cycle as a function of the terminal alkoxy chain length of *i*O-PPQOMe, *i* = 3–8, compounds.

In the series of *i*O-PPQOMe compounds, the mesogenic core is composed of a long linear biphenyl group and a kinked quinoline [23]. All *i*O-PPQOMe, *i* = 3–8, compounds exhibited the enantiotropic nematic phase in a high temperature range of 200–350 °C, with wide mesomorphic lengths in the range of 90–140 °C, as shown in Figure 4. The nematic mesomorphic phase reached the decomposition temperature when the alkoxy chain was short, that is, *i* = 3 and 4. In addition, a narrow crystalline mesophase (CrX) appeared on a second heating scan of *8*O-PPQOMe.

Apparently, a mesogenic core with a high aspect ratio and containing a kinked moiety, such as quinoline, benefits not only the generation of the nematic phase but also the stability of the mesomorphic range. Unlike liquid crystal molecules with a straight linear mesogenic core [36,37], polymesomorphism did not appear in the *i*O-PPQOMe series with an increasing alkoxy chain length. The authors believe that the kinked moiety (quinoline) maintains the persistent appearance of the enantiotropic nematic phase as the alkoxy chain length increases in the series of *i*O-PPQOMe compounds. Hysteresis for one kinked structure in *i*O-PPQOMe were considerably milder than those for two-kinked structures in *n*O-NpQOMe compounds.

Following the decreasing trend of the melting points form 210.6 to 190.2 °C (difference: 20.4 °C) and freezing points from 191.5 to 176.2 °C (difference: 15.3 °C), *T*_NI_ (or *T*_IN_) decreased from 311 to 263.1 °C (difference: 47.9 °C) as the alkoxy chain length increased. These results again suggest that the soft alkoxy chain that suppresses the crystallization in this study seems functioning as “impurity” within the three-dimension crystalline/liquid-crystalline lattice structure consisting of anisotropic hard cores. The mesogenic core containing biphenyl and kinked quinoline moieties is responsible for the collapsing and formation processes of crystal-to-nematic and nematic-to-isotropic transitions. By contrast, although soft alkoxy chains participated in these collapsing and formation processes, they played a minor role. However, the variation in the nematic-to-isotropic collapsing (formation) transition with an increasing in the alkoxy chain, 47.9 °C, was considerably wider than that in melting and freezing transitions, 20.4, and 15.3 °C, respectively.

The ΔS_IN_/R values for the series of *i*O-PPQOMe compounds were 0.10, 0.13, 0.13, and 0.11 for *n* = 5–8, respectively. Again, these low values for the isotropic-to-nematic transitional entropy can be attributed to the molecular biaxiality originating from the kinked quinoline moiety.

**Figure 5:**
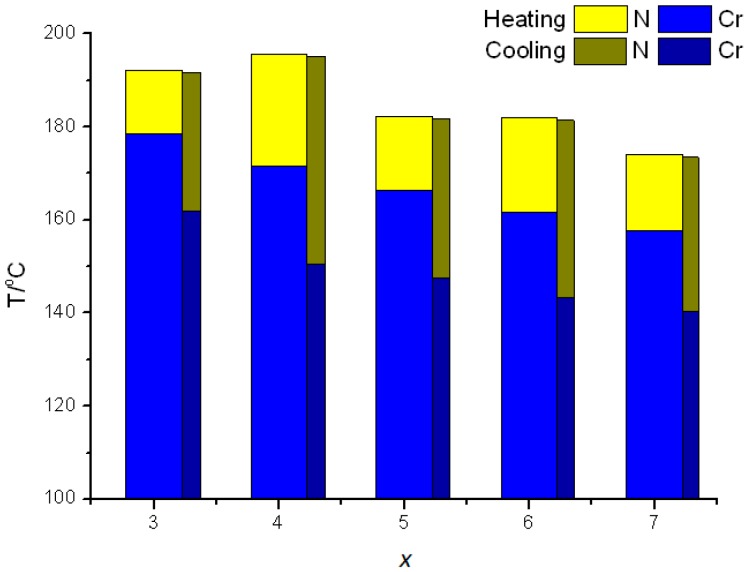
Plot of transition temperatures of heating and cooling cycle as a function of the terminal alkoxy chain length for *x*O-NpQMe, *x* = 3–7, compounds.

Compared with the series of *n*O-NpQOMe compounds with a polar methoxy end group, the series of *x*O-NpQMe compounds had a nonpolar methyl end group [38]. Both series had the same type of two consecutive kinked mesogenic cores (naphthalene and quinoline) along their molecular long axis. All *x*O-NpQMe (*x* = 3–7) compounds exhibited only the enantiotropic nematic phase at the medium–high temperature range 140.2–192.2 °C, which is somewhat lower than that of *n*O-NpQOMe, *n* = 3–7, compounds, 163.2–234.2 °C, as shown in Figure 5. Independent of the end-groups, both series exhibited only the enantiotropic nematic phase. It is evident that the two consecutive kinked, asymmetrical mesogenic moieties, naphthalene and quinoline, play a major role in the formation of enantiotropic nematic phase. The hard mesogenic core consisting of naphthalene and quinoline determines the mesophase type (nematic), the way mesophase appearing (enantiotropic) and the approximate range (~20 °C at heating and ~40 °C at cooling) and range ratio (heating/cooling = 1/2) of the mesophase.

The nematic phase lengths of *x*O-NpQMe, *x* = 3–7, compounds during heating and cooling were in a range of 13.7–24.1 and 29.7–44.6 °C, respectively, which are comparable with and slightly narrower than those of *n*O-NpQOMe, *n* = 3–7, compounds, 16.6–26.4 and 37.0–55.1 °C, respectively. Furthermore, hysteresis was observed in the series of *x*O-NpQMe compounds, corroborating the evidence that the kinked, asymmetrical mesogenic core structure prevents nucleation during crystallization and delays the freezing process.

The odd–even effect can be clearly observed from the nematic phase lengths for the series of *x*O-NpQMe compounds. On cooling, the nematic phase lengths were 44.6 and 38.1 °C when *x* = 4 and 6 and 29.8, 34.4, and 33.0 °C when *x* = 3, 5, and 7, respectively. The same phenomenon was observed for heating (24.1 and 20.3 °C when *x* = 4 and 6 and 13.6, 16.0, and 16.5 °C when *x* = 3, 5, and 7, respectively). By contrast, the nematic phase lengths of *n*O-NpQOMe, *n* = 3–8, compounds were independent of the odd-even effect. Apparently, the nonpolar methyl end group reduced the amount of dimeric aggregation in the nematic phase, thus making the molecules of *x*O-NpQMe in the mesophase more susceptible to the odd–even effect.

In addition, the odd-even effect of *T*_NI_ (*T*_IN_) was observed in the beginning of the *x*O-NpQMe series; in which *x* = 3, 4, and 5 exhibited *T*_NI_ (*T*_IN_) values of 192.1 (191.4), 195.6 (194.9), and 182.3 (181.5) °C, respectively. However, *T*_NI_ (*T*_IN_) decreased with an increase in the chain length when *x* ≥ 5; the *T*_NI_ (*T*_IN_) values were 182.0 (181.1), and 174.0 (173.2) °C when *x* = 6 and 7, respectively. This corroborates the notion that a mesogen with a nonpolar end group is more susceptible to the odd–even effect of the soft alkoxy chains, particularly when the alkoxy tails are short.

As mentioned in the series of *n*O-NpQOMe, *n* = 3–7, compounds, the melting and freezing points decreased with an increase in the alkoxy chain length. By contrast, in the series of *x*O-NpQMe, *x* = 3–7, compounds, the melting and freezing points decreased from 178.4 to 157.6 °C (difference: 20.8 °C) and from 161.7 to 140.2 °C (difference: 21.5 °C), respectively, with an increase in the alkoxy chain length. An increased temperature difference in the melting (29.3 *vs.* 20.8 °C) and freezing processes (23.5 *vs.* 21.5 °C) was observed for these two series. This suggests that the polar methoxy end group enhances the variation in melting and freezing processes as the alkoxy chain length increases.

## 3. Experimental Section

### 3.1. General

The chemical structures of these compounds were analyzed by ^1^H- and ^13^C-NMR spectra using a Bruker AC 300 spectrometer (Bruker Corporation, Billerica, MA, USA). Infrared (IR) spectra were recorded on a Perkin-Elmer 1600 Series spectrometer (Perkin-Elmer, Norwalk, CT, USA). The purity of these compounds was checked by thin-layer chromatography and further confirmed by elemental analysis.

Mesophases were chiefly identified by microscopic texture of samples sandwiched between two glass plates under a polarizing optical microscope (POM; Olympus BH-2, Two Corporate Center Drive, Melville, NY, USA) equipped with a Mettler FP90/FP82HT hot stage (Mettler, Columbus, OH, USA). Phase transition temperatures and their corresponding transition enthalpies were determined by differential scanning calorimetry (DSC), using a Perkin-Elmer DSC 7 calorimeter at a scanning rate of 5 °C·min^−1^. The program consists of first heating and followed by repeated cooling and heating cycles. Data from the repeated cycles were found to be identical to those of the first cooling and second heating. Therefore, only those data from the first cooling and second heating cycles are reported.

### 3.2. Synthesis

The starting materials, 2-bromo-6-hydroxynaphthalene, and 6-methoxyquinoline were purchased from Aldrich Chemical Co. (Saint Louis, MO, USA) and used as received. Phenyl chloroformate and *n*-bromoalkanes were distilled under an inert nitrogen atmosphere immediately before use. Anhydrous organic solvents, toluene and tetrahydrofuran (THF), were first heated at reflux over sodium and then distilled under nitrogen before use. Column chromatography was carried out with silica gel (MN Kieselgel 60, 70–230 mesh; Duren, Germany). The purity of the compounds was checked by thin-layer chromatography and further confirmed by elemental analysis. The synthesis of 2-(6-alkoxynaphthalen-2-yl)-6-methoxyquinolines was carried out according to the synthetic methods outlined in Scheme 1.

Representative Procedure for the Homologues of 2-(6-Alkoxynaphthalen-2-yl)-6-methoxyquinolines (*n*O-NpQOMe, *n* = 3–8)

The entire synthetic procedures were completed in a short two-step process with overall yields in the range of 53%–62% (Table 1). For 4O-NpQOMe in **3**, to a solution of 2-bromo-6-butoxynaphthalene (10 mmol) in THF (20 mL), freshly dried magnesium granules (11 mmol) were added under an inert nitrogen atmosphere for about half an hour. The Grignard solution **1** was then slowly added by a syringe into a preformed solution of 6-methoxyquinolinium chloride **2**, which was prepared from phenyl chloroformate (10 mmol) and 6-methoxyquinoline (10 mmol) in dry THF (20 mL) at −20 °C for half an hour. The resulting solution was heated slowly to room temperature and stirred for another 8 h. After the solvent THF was evaporated, the residue was extracted with Et_2_O. The organic layer was further washed once with 20% NH_4_Cl solution and twice with distilled water and brine and dried with magnesium sulfate. For 4O-NpQOMe in **4**, to a solution of dry toluene (20 mL) and compound **3** (10 mmol), about 1.3 equivalent *o*-chloranil was added. The reaction mixture was heated to reflux for about 3 h under inert nitrogen atmosphere and then quenched by adding 1 N NaOH (25 mL) and Et_2_O (25 mL) and filtered through Celite (Acros Organics, Duren, Germany). Normal aqueous work-up and isolation with column chromatography (methylene chloride: hexane = 2:1) afford an overall two-step reaction with good yield of 2-(6-butoxynaphthalen-2-yl)-6-methoxyquinoline **4** (60%). The crude products of **4** were further purified by recrystallisation from a mixed solvent of methylene chloride and ethyl acetate. The other *n*O-NpQOMe homologues were synthesized essentially by the same procedure as described above for the *n* = 4 homologue. All compounds gave satisfactory ^1^H-NMR, ^13^C-NMR, IR and elemental analysis results as discussed in the following section.

2-(6-Propoxynaphthalen-2-yl)-6-methoxyquinoline (3O-NpQOMe). ^1^H-NMR (CDCl_3_): δ 8.51 (s, 1H, naphthalene), 8.28 (d, 1H, *J* = 8.1 Hz, naphthalene), 8.08–8.15 (m, 2H, quinoline), 7.97 (d, 1H, *J* = 9 Hz, naphthalene), 7.84–7.89 (m, 2H, 1 in naphthalene 1 in quinoline), 7.39 (dd, 1H, *J*_1_ = 2.4 Hz, *J*_2_ = 9 Hz, quinoline), 7.18–7.26 (m, 2H, naphthalene), 7.11 (d, 1H, *J* = 2.1 Hz, quinoline), 4.08 (t, 2H, *J* = 6.6 Hz, -CH_2_), 3.96 (s, 2H, -OCH_3_), 1.90 (sext, 2H, *J* = 6.6 Hz, -CH_2_), 1.10 (t, 3H, *J* = 7.6 Hz, -CH_3_). ^13^C-NMR (CDCl_3_): ppm 157.9, 157.7, 155.2, 144.6, 135.6, 135.1, 134.9, 131.2, 130.3, 129.0, 128.2, 127.4, 126.6, 125.5, 122.4, 119.6, 119.4, 106.5, 105.2, 69.7, 55.7, 22.7, 10.8. IR (KBr): cm^−1^ 3032 (aromatic C–H stretch), 2937 (aliphatic C–H asymmetric stretch), 2879 (aliphatic C–H asymmetric stretch), 1623 (ring stretch), 1596 (ring stretch), 1466 (C=N stretch), 1043 (asymmetric C–O–C stretch), 1025 (symmetric C–O–C stretch), 819 (out-of-plane C–H bend), 807 (out-of-plane C–H bend). Anal. calcd for C_23_H_21_NO_2_: C, 80.44; H, 6.16; N, 4.08.

2-(6-Butoxynaphthalen-2-yl)-6-methoxyquinoline (4O-NpQOMe). ^1^H-NMR (CDCl_3_): δ 8.50 (s, 1H, naphthalene ), 8.29 (dd, 1H, *J*_1_ = 1.8 Hz, *J*_2_ = 8.7 Hz, naphthalene), 8.08–8.15 (m, 2H, quinoline), 7.97 (d, 1H, *J* = 8.4 Hz, naphthalene), 7.84–7.89 (m, 2H, 1 in naphthalene 1 in quinoline), 7.39 (dd, 1H, *J*_1_ = 9 Hz, *J*_2_=2.7 Hz, quinoline), 7.17–7.21 (m, 2H, naphthalene), 7.11 (d, 1H, *J* = 2.7 Hz, quinoline), 4.12 (t, 2H, *J* = 6.6 Hz, -CH_2_), 3.96 (s, 1H, -OCH_3_), 1.86 (quin, 2H, *J* = 6.3 Hz, -CH_2_), 1.56 (m, 2H, -CH_2_), 1.02 (t, 3H, *J* = 7.5 Hz, -CH_3_). ^13^C-NMR (CDCl_3_): ppm 158.6, 158.3, 155.8, 145.2, 136.2, 135.8, 135.6, 131.9, 131.0, 129.7, 128.8, 128.0, 127.2, 126.2, 123.0, 120.2, 120.0, 107.2, 105.9, 68.5, 56.4, 32.1, 20.1, 14.7. IR (KBr): cm^−1^ 3029 (aromatic C–H stretch), 2932 (aliphatic C–H asymmetric stretch), 2880 (aliphatic C–H asymmetric stretch), 1625 (ring stretch), 1597 (ring stretch), 1467 (C=N stretch), 1043 (asymmetric C–O–C stretch), 1025 (symmetric C–O–C stretch), 817 (out-of-plane C–H bend), 808 (out-of-plane C–H bend). Anal. calcd for C_24_H_23_NO_2_: C, 80.64; H, 6.49; N, 3.92. C, Found: 80.75; H, 6.42; N, 3.55.

2-(6-Pentoxynaphthalen-2-yl)-6-methoxyquinoline (5O-NpQOMe). ^1^H-NMR (CDCl_3_): δ 8.50 (s, 1H, naphthalene), 8.29 (dd, 1H, *J*_1_ = 8.7 Hz, *J*_2_ = 1.5 Hz, naphthalene), 8.08–8.14 (m, 2H, quinoline), 7.96 (d, 1H, *J* = 8.7 Hz, naphthalene), 7.84–7.87 (m, 2H, 1 in naphthalene 1 in quinoline), 7.39 (dd, 1H, *J*_1_ = 9 Hz, *J*_2_ = 2.7 Hz, quinoline), 7.17–7.21 (m, 2H, quinoline), 7.10 (d, 1H, *J* = 2.7 Hz, quinoline), 4.11 (t, 2H, *J* = 6.6 Hz, -CH_2_), 3.95 (s, 3H, -OCH_3_), 1.88 (quin, 2H, *J* = 6.6 Hz, -CH_2_), 1.47 (m, 4H, -CH_2_), 0.96 (t, 2H, *J* = 7.2 Hz, -CH_3_). ^13^C-NMR (CDCl_3_): ppm 157.9, 157.7, 144.6, 135.6, 135.1, 134.9, 131.2, 130.3, 129.0, 128.2, 127.4, 126.6, 125.5, 122.4, 119.6, 119.4, 106.5, 105.2, 79.27, 68.2, 55.7, 29.1, 28.4, 22.6, 14.2. IR (KBr): cm^−1^ 3031 (aromatic C–H stretch), 2932 (aliphatic C–H asymmetric stretch), 2880 (aliphatic C–H asymmetric stretch), 1625 (ring stretch), 1596 (ring stretch), 1467 (C=N stretch), 1043 (asymmetric C–O–C stretch), 1025 (symmetric C–O–C stretch), 819 (out-of-plane C–H bend), 810 (out-of-plane C–H bend). Anal. calcd for C_25_H_25_NO_2_: C, 80.83; H, 6.78; N, 3.77. 

2-(6-Hexoxynaphthalen-2-yl)-6-methoxyquinoline (6O-NpQOMe). ^1^H-NMR (CDCl_3_): δ 8.50 (s, 1H, naphthalene), 8.29 (dd, 1H, *J*_1_ = 8.7 Hz, *J*_2_ = 1.5 Hz, naphthalene), 8.08-8.14 (m, 2H, quinoline), 7.96 (d, 1H, *J* = 8.7 Hz, naphthalene), 7.84–7.88 (m, 2H, 1 in naphthalene 1 in quinoline), 7.40 (dd, 1H, *J*_1_ = 9 Hz, *J*_2_ = 2.7 Hz, quinoline), 7.17–7.21 (m, 2H, quinoline), 7.10 (d, 1H, *J* = 2.7 Hz, quinoline), 4.10 (t, 2H, *J* = 6.6 Hz, -CH_2_), 3.95 (s, 3H, -OCH_3)_, 1.87 (quin, 2H, *J* = 6.9 Hz, -CH_2_), 1.42 (m, 6H, -CH_2_), 0.93 (t, 2H, *J* = 7.2 Hz, -CH_3_). ^13^C-NMR (CDCl_3_): 157.9, 157.7, 155.2, 144.6, 135.6, 135.1, 134.9, 131.2, 130.3, 129.0, 128.1, 127.4, 126.6, 125.5, 122.4, 119.6, 119.4, 106.5, 105.2, 68.2, 55.7, 31.8, 29.4, 25.9, 22.8, 14.2. IR (KBr): cm^−1^ 3034 (aromatic C–H stretch), 2928 (aliphatic C–H asymmetric stretch), 2858 (aliphatic C–H asymmetric stretch), 1625 (ring stretch), 1597 (ring stretch), 1468 (C=N stretch), 1044 (asymmetric C–O–C stretch), 1026 (symmetric C–O–C stretch), 820 (out-of-plane C–H bend), 807 (out-of-plane C–H bend). Anal. calcd for C_26_H_27_NO_2_: C, 81.01; H, 7.06; N, 3.63.

2-(6-Heptoxynaphthalen-2-yl)-6-methoxyquinoline (7O-NpQOMe). ^1^H-NMR (CDCl_3_): δ 8.51 (d, 1H, *J* = 1.5 Hz, naphthalene), 8.29 (dd, 1H, *J*_1_ = 9 Hz, *J*_2_ = 2.4 Hz, naphthalene), 8.08–8.12 (m, 2H, quinoline), 7.97 (d, 1H, *J* = 8.4 Hz, naphthalene), 7.84–7.89 (m, 2H, 1 in quinoline 1 in naphthalene), 7.39 (dd, 1H, *J*_1_ = 9 Hz, *J*_2_ = 3 Hz, quinoline), 7.17–7.21 (m, 2H, naphthalene), 7.11 (d, 1H, *J* = 3 Hz, quinoline), 4.10 (t, 2H, *J* = 6.3 Hz, -CH_2_), 3.96 (s, 3H, -OCH_3_), 1.87 (quin, 2H, *J* = 6.6 Hz, -CH_2_), 1.25–1.54 (m, 8H, -CH_2_), 0.91 (t, 2H, *J* = 7.2 Hz, -CH_3_). ^13^C-NMR (CDCl_3_): 158.0, 157.8, 155.2, 144.6, 135.7, 135.2, 135.0, 131.3, 130.4, 129.1, 128.2, 127.5, 126.6, 125.6, 122.5, 119.7, 119.5, 106.6, 105.3, 68.3, 55.8, 32.0, 29.5, 29.3, 26.3, 22.8, 14.3. IR (KBr): cm^−1^ 3031 (aromatic C–H stretch), 2924 (aliphatic C–H asymmetric stretch), 2859 (aliphatic C–H asymmetric stretch), 1621 (ring stretch), 1597 (ring stretch), 1466 (C=N stretch), 1041 (asymmetric C–O–C stretch), 1027 (symmetric C–O–C stretch), 819 (out-of-plane C–H bend), 806 (out-of-plane C–H bend). Anal. calcd for C_27_H_29_O_2_: C, 81.17; H, 7.32; N, 3.51.

2-(6-Octoxynaphthalen-2-yl)-6-methoxyquinoline (8O-NpQOMe). ^1^H-NMR (CDCl_3_): δ 8.50 (s, 1H, naphthalene), 8.30 (d, 1H, *J* = 9 Hz, naphthalene), 8.06–8.15 (m, 2H, quinoline), 7.96 (d, 1H, *J* = 8.7 Hz, naphthalene),7.84–7.89 (m, 2H, 1 in naphthalene 1 in quinoline), 7.39 (dd, 1H, *J*_1_ = 9 Hz, *J*_2_ = 1.8 Hz, quinoline), 7.17–7.20 (m, 2H, quinoline), 7.11 (d, 1H, *J* = 2.4 Hz, quinoline), 4.10 (t, 2H, *J* = 6.6 Hz, -CH_2_), 3.95 (s, 3H, -OCH_3_), 1.85 (quin, 2H, *J* = 6.9 Hz, -CH_2_), 1.28 (m, 10H, -CH_2_), 0.88 (d, 3H, *J* = 6.6 Hz, -CH_3_). ^13^C-NMR (CDCl_3_): 157.9, 157.7, 155.2, 144.6, 135.6, 135.2, 134.9, 131.2, 130.3, 129.0, 128.2, 127.4, 126.6, 125.5, 122.4, 119.6, 119.4, 106.5, 105.2, 68.2, 55.7, 45.5, 32.0, 29.5, 29.4, 26.3, 22.8, 14.3. IR (KBr): cm^−1^ 3029 (aromatic C–H stretch), 2924 (aliphatic C–H asymmetric stretch), 2855 (aliphatic C–H asymmetric stretch), 1625 (ring stretch), 1597 (ring stretch), 1469 (C=N stretch), 1044 (asymmetric C–O–C stretch), 1025 (symmetric C–O–C stretch), 820 (out-of-plane C–H bend), 807 (out-of-plane C–H bend). Anal. calcd for C_28_H_31_NO_2_: C, 81.32; H, 7.56; N, 3.39.

## 4. Conclusions

Nematic phase, a semi-homogeneous phase, is indeed a technologically important phase. With suitable design of molecules combining with their self-assembly behavior, nematic phase can be engineered and integrated into many current thin-film and semiconductor solid-state devices. Thus, understanding of the intrinsic factors forming nematic phase is crucial to their future application.

In this study, a new series of two consecutive kinked liquid crystalline compounds, 2-(6-alkoxynaphthalen-2-yl)-6-methoxyquinolines (*n*O-NpQOMe, *n* = 3–8) was synthesized through a convenient and a short two-step route, and the thermotropic properties of these compounds were further examined. Fair to satisfactory two-step overall yields of 53%–62% were obtained.

All *n*O-NpQOMe, *n* = 3–8, compounds exhibited the enantiotropic nematic phase. Supercooling phenomenon of the nematic phase was observed for these two-kinked liquid crystalline compounds. In addition, the odd–even effect, the aspect ratio of the mesogenic core, (polar and nonpolar) end groups, the number of kinks, *T*_NI_ (*T*_IN_) values, the nematic phase lengths, and melting and freezing points were studied and compared among the four series of compounds, *n*O-NpQOMe, *n* = 3–8, *i*O-PPQOMe, *i* = 3–8, *m*O-PQOMe, *m* = 3–8, and *x*O-NpQMe, *x* = 3–7. Thus, the structural factors of the liquid crystal molecules that affect the nematic phase were manifested. In all, the kinked mesogenic core structure can be used to produce nematic and only nematic phase.
